# Mobile Phone Addiction and Sleep Quality Among Children and Adolescents: Unraveling the Health Consequences

**DOI:** 10.7759/cureus.102460

**Published:** 2026-01-28

**Authors:** Ghizal Fatima, Vani Shukla, Aminat Magomedova, Rasul Magomedov

**Affiliations:** 1 Department of Biotechnology and Chronobiology Unit, Era University, Lucknow, IND; 2 Department of Nutrition, Era University, Lucknow, IND; 3 Department of Population, Lomonosov Moscow State University, Moscow, RUS; 4 Department of Computational Mathematics and Cybernetics, Lomonosov Moscow State University, Moscow, RUS

**Keywords:** children, cognitive development, digital habits, mobile phone addiction, sleep quality

## Abstract

The pervasive use of mobile phones has emerged as a growing public health concern, particularly among children and adolescents, with increasing evidence linking excessive screen exposure to sleep disturbances. This study was conducted as a narrative review of the existing literature to examine the association between mobile phone addiction and sleep quality in children and adolescents aged five to 18 years. Relevant peer-reviewed articles were identified through electronic database searches, including PubMed, Scopus, and Google Scholar. Studies published in English that investigated mobile phone use, screen exposure, sleep parameters, circadian rhythm disruption, or related psychological outcomes in pediatric and adolescent populations were included. Studies focusing exclusively on adults, clinical sleep disorders unrelated to screen exposure, or non-human models were excluded. The review synthesizes findings related to physiological, psychological, and behavioral mechanisms underlying sleep disturbances. The reviewed evidence indicated that prolonged and inappropriate mobile phone use, particularly during evening and pre-bedtime hours, disrupts circadian rhythms primarily through blue light-induced suppression of melatonin, resulting in delayed sleep onset, reduced sleep duration, and impaired sleep quality. Psychological factors, including anxiety, hyperarousal, and fear of missing out (FOMO), further exacerbate sleep disturbances. Poor sleep quality among children and adolescents is consistently associated with adverse outcomes, including impaired cognitive performance, emotional dysregulation, weakened immune function, and increased risk of obesity. This review highlights mobile phone addiction as a significant modifiable risk factor for poor sleep quality in children and adolescents. The findings underscore the urgent need for multilevel interventions involving parents, educators, healthcare professionals, and policymakers. Strategies such as structured screen time limits, digital literacy education, and promotion of healthy sleep hygiene and alternative recreational activities are essential to mitigate the negative impact of excessive mobile phone use on sleep and overall well-being in the digital age.

## Introduction and background

Mobile phone addiction has become a growing concern, particularly among children and adolescents. With the increasing reliance on smartphones for communication, entertainment, and education, children are spending more time on their devices. This excessive usage can lead to several health issues, particularly affecting sleep quality. The relationship between mobile phone addiction and sleep disturbances is alarming, as poor sleep quality can impact children's cognitive development, emotional regulation, and overall well-being.

This review explores the growing issue of mobile phone addiction among children and adolescents, and its significant impact on sleep quality. With smartphones becoming an integral part of daily life, the boundaries between necessary use and excessive dependence have blurred, particularly for younger users. The overuse of mobile devices, especially at night, has been linked to sleep disturbances, delayed bedtimes, and reduced sleep duration. As sleep is essential for physical and cognitive development, understanding the health implications of mobile phone addiction on children's sleep patterns is crucial for parents, educators, and healthcare providers [1].

Mobile phone addiction, also known as problematic mobile phone use (PMPU), has increasingly become a public health concern among children in recent years. With the proliferation of smartphones and internet access, children are being introduced to mobile devices at a younger age, making them more susceptible to addiction [2]. Research indicates that mobile phone addiction is characterized by compulsive usage patterns, withdrawal symptoms when deprived of access, and an overall disruption to daily life, including school performance and social interactions. This problematic behavior is often driven by the accessibility of online gaming, social media, and instant messaging, which provide instant gratification and foster dependency [3].

**Figure 1:**
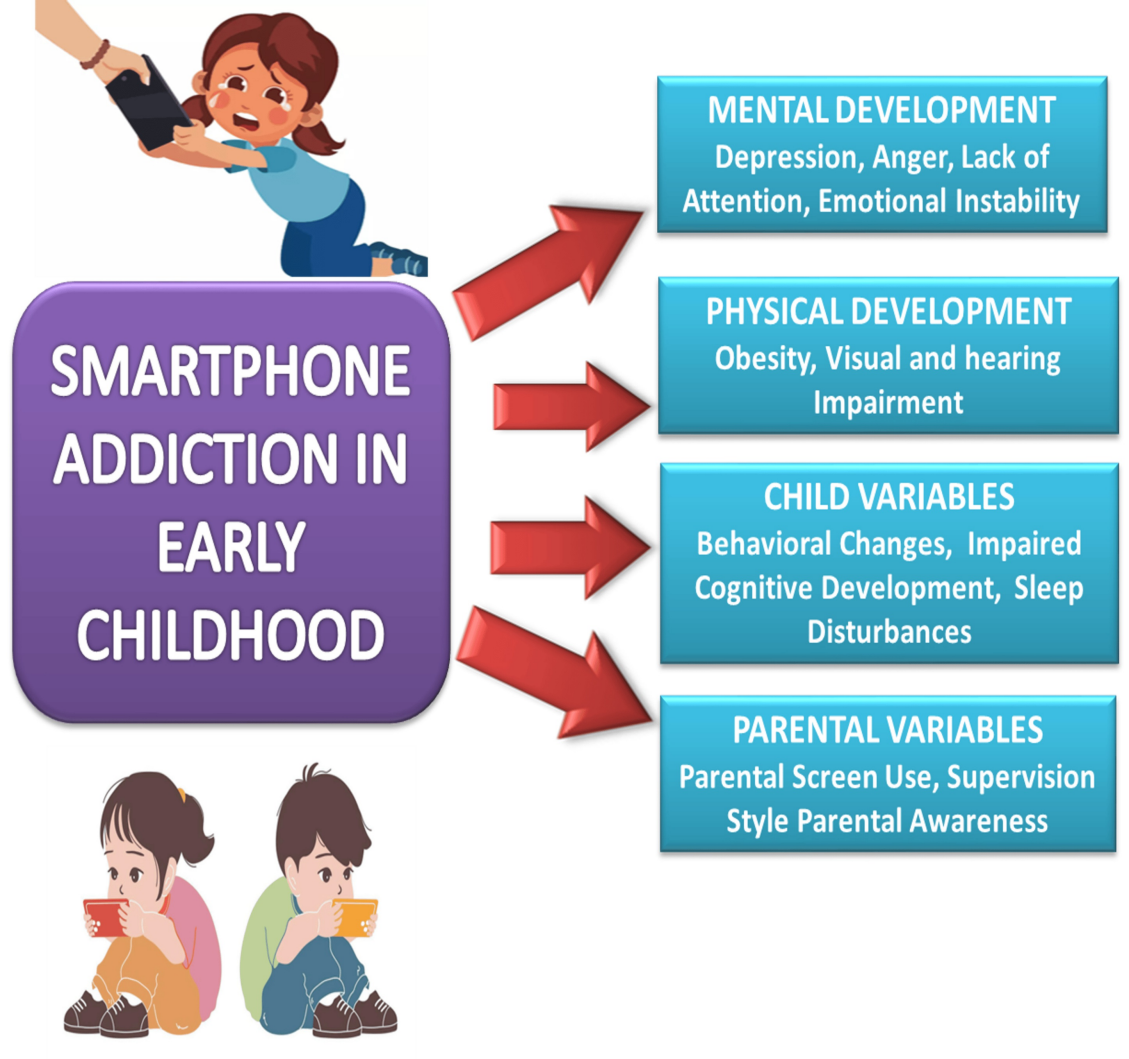
A model of smartphone addiction in early childhood Image credit: Created by Fatima G using Microsoft PowerPoint (Microsoft Corp., Redmond, WA, USA).

Children are particularly vulnerable to mobile phone addiction due to their developing cognitive control and emotional regulation abilities. The addictive design of mobile applications through notifications, likes, and comments stimulates the brain's reward system, making it difficult for young users to limit their screen time. Numerous detrimental psychological and physiological effects, such as anxiety, depression, decreased physical activity, and sleep difficulties, have been connected to this. Before going to bed, using a mobile phone has been demonstrated to disrupt the quality of sleep by postponing the onset of sleep and shortening the duration of sleep. Screen blue light reduces the production of melatonin, a hormone that controls sleep cycles, which results in less restful sleep and daytime cognitive decline [4]. Recent studies have demonstrated that the long-term consequences of mobile phone addiction in children include difficulties in attention regulation, lower academic achievement, and strained interpersonal relationships [5]. Addressing this issue requires comprehensive strategies involving parents, educators, and healthcare providers. It is essential to establish clear guidelines on screen time, promote digital literacy, and encourage alternative recreational activities to balance children's digital and physical lives [6,7]. Figure 1 highlights the consequences of smartphone addiction in early childhood, affecting mental and physical development, behavior, and the impact of parental influence.

It leads to issues like depression, anger, obesity, visual and hearing impairments, cognitive delays, and sleep disturbances. Parental screen habits, supervision, and awareness also play a crucial role in shaping outcomes.

Importance of sleep quality in childhood development

Table 1 summarizes key studies highlighting the importance of sleep quality in childhood development.

**Table 1:** Studies assessing the importance of sleep quality in childhood development

Key findings	Focus area
Sleep supports brain maturation, neural plasticity, memory consolidation, and learning processes.	Cognitive development [8]
Poor sleep quality is associated with attention, memory, and executive functioning difficulties.	Cognitive performance [9]
Inadequate sleep in children leads to emotional dysregulation, increased irritability, and mood swings.	Emotional regulation [10]
Sleep deprivation disrupts hormone balance (cortisol, melatonin), affecting stress responses and mental health.	Stress responses and mental health [11]
Disrupted sleep is linked to impaired immune response, slower growth, and increased obesity risk due to hormonal imbalances.	Physical development and obesity [12]

Each study focuses on how sleep impacts physical, cognitive, emotional, and mental well-being.

## Review

Mobile phone addiction: a growing concern

Conceptualizing Mobile Phone Addiction

Mobile phone addiction, also referred to as PMPU, is increasingly recognized as a behavioral addiction that exhibits symptoms similar to other addictive behaviors, such as gambling or substance abuse [13]. This addiction is characterized by excessive and compulsive use of mobile devices that interferes with daily activities, responsibilities, and social relationships. Unlike general mobile phone usage, addiction implies a loss of control, where individuals feel compelled to engage with their phones despite negative consequences such as social isolation, poor academic performance, or sleep disturbances [14]. The addictive nature of mobile phones is largely due to the vast array of applications that provide instant gratification through social media, games, and messaging platforms, all of which stimulate the brain's reward system, particularly the release of dopamine [14]. From a psychological perspective, mobile phone addiction is linked to both impulsive behavior and emotional dysregulation, particularly among younger populations. Research has shown that individuals addicted to mobile phones tend to exhibit higher levels of anxiety, depression, and stress [15]. Moreover, the constant connectivity provided by mobile devices leads to a phenomenon known as nomophobia, or the fear of being without a mobile phone, which further perpetuates dependency [16]. Mobile phone addiction is thus not merely an overuse issue but is a complex condition that affects emotional, cognitive, and social functioning.

Prevalence and Demographics

Table 2 summarizes key studies on the prevalence and demographics of mobile phone addiction, particularly among adolescents.

**Table 2:** Studies evaluating the key factors influencing mobile phone addiction across demographics and regions

Aspect	Details
Global prevalence [17]	Mobile phone addiction affects 25-33% of adolescents globally, with higher rates in specific regions.
Geographic variations [18]	Mobile phone addiction is particularly high in China, South Korea, and the United States, reflecting high digital adoption.
Age and susceptibility [19]	Younger individuals are more prone to mobile phone addiction due to increased engagement with social media and online gaming.
Gender differences [20]	Males are more likely to develop addiction related to gaming, while females tend to overuse mobile phones for social networking.
Socioeconomic status [21]	Individuals from lower socioeconomic backgrounds face higher risks of mobile phone addiction due to limited alternatives and increased stress.

These studies show how factors such as age, gender, and socioeconomic status influence the likelihood of developing mobile phone addiction, as well as the global variations in its prevalence.

Risk Factors for Addiction

Several risk factors contribute to the development of mobile phone addiction, particularly among children and adolescents. One of the primary risk factors is the reward system built into mobile applications, especially social media platforms and online games. These platforms use notifications, likes, comments, and instant messaging to provide users with immediate gratification, triggering dopamine release in the brain, and reinforcing compulsive behavior [14]. This neurological reward system is particularly effective in younger populations, whose self-regulation and impulse control are still developing [22].

Personality traits also play a significant role in mobile phone addiction. Individuals with high levels of impulsivity, emotional instability, and anxiety are more prone to developing addictive behaviors with their mobile devices [23]. For example, those who struggle with emotional regulation may use their phones to seek comfort or distraction, further entrenching the addictive cycle [15]. Social anxiety and loneliness have been identified as predictors of mobile phone addiction, with individuals turning to their devices as a means of avoiding face-to-face interactions and coping with social isolation [24].

One more critical risk factor is screen time, particularly the use of mobile phones before bedtime. Sleep disturbances are both a symptom and a risk factor for mobile phone addiction, as individuals may use their phones to pass time during sleepless hours, further exacerbating their dependence. Parental monitoring and peer influence are significant social factors contributing to mobile phone addiction. Adolescents with less parental supervision are more likely to develop problematic phone use, as they lack the guidance needed to set healthy boundaries for screen time [23]. Likewise, peer pressure to remain constantly connected through social media can lead to excessive phone use and feelings of anxiety when disconnected [25].

Sleep quality in children: an essential component of health

Understanding Sleep Quality

**Figure 2:**
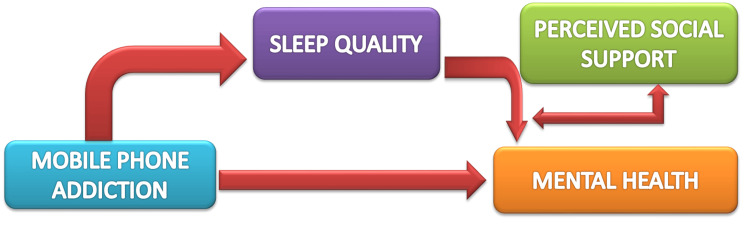
Mobile phone addiction and mental health: the role of sleep quality and perceived social support Image credit: Created by Fatima G using Microsoft PowerPoint (Microsoft Corp., Redmond, WA, USA).

Sleep quality is a multifaceted concept that goes beyond simply the duration of sleep; it encompasses various elements such as sleep onset latency, sleep maintenance, sleep efficiency, and the subjective experience of feeling rested upon waking. In children, sleep quality is an essential determinant of physical, cognitive, and emotional health, influencing everything from brain development to mood regulation [26]. Poor sleep quality is often characterized by difficulties in falling asleep, frequent awakenings during the night, and a lack of deep, restorative sleep [27]. While sleep duration is often used as a proxy for sleep quality, it is the depth and continuity of sleep that play a crucial role in the brain's ability to consolidate memories and support cognitive functions such as attention, learning, and emotional regulation (Figure 2) [9].

The importance of sleep quality in childhood cannot be overstated, as this period is critical for growth and development. Research has shown that children who experience poor sleep quality are more likely to exhibit behavioral problems, emotional dysregulation, and difficulties in school performance [12]. Additionally, poor sleep has been linked to physical health issues such as impaired immune function and an increased risk of obesity [28]. Factors such as environmental stressors, inconsistent bedtime routines, and the use of electronic devices before sleep can all negatively impact sleep quality, making it a growing concern for parents and healthcare professionals [29,30].

Sleep Patterns in Children

Table 3 describes the key studies on sleep patterns in children, and shows the variations in sleep needs based on age, the developmental changes in sleep cycles, and the potential impacts of disrupted sleep.

**Table 3:** Studies evaluating sleep patterns in children REM: Rapid eye movement.

Age group	Sleep duration and pattern	Key findings
Newborns (0-3 months) [31]	14-17 hours per day, fragmented into shorter intervals with frequent awakenings.	Sleep is distributed across multiple naps throughout the day and night, with a larger proportion of REM sleep.
Preschool-aged (3-5 years) [31]	10-13 hours per night.	Sleep consolidates into longer periods, with regular bedtime routines improving sleep quality.
School-aged (6-12 years) [31]	9-11 hours per night.	More stable deep sleep cycles develop, which are essential for cognitive and physical development.
Adolescents (13-18 years) [32]	8-10 hours per night.	Sleep patterns may be disrupted by environmental factors such as electronic device usage, leading to sleep deprivation.
Infants (0-1 year) [33]	A high proportion of REM sleep.	REM sleep is essential for brain development and consolidating neural connections.
Impact of device use (all ages) [34]	Sleep disruptions lead to delayed sleep onset, frequent night awakenings, and daytime sleepiness.	The use of electronic devices at night can cause sleep disturbances and impair sleep quality.
Cognitive and behavioral impact [35]	Irregular sleep patterns lead to attention difficulties, emotional dysregulation, and poor academic performance.	Sleep disruption affects cognitive development, emotional control, and behavior.
Physical health impact [36]	Poor sleep increases the risk of obesity and weakens immune function, disrupting metabolism and hormonal regulation.	Sleep deprivation negatively affects physical health and increases the risk of chronic health conditions.

Consequences of Poor Sleep Quality

Poor sleep quality in children has far-reaching consequences, impacting physical, cognitive, emotional, and social development. One of the most immediate effects is on cognitive functioning, as sleep is essential for memory consolidation, attention, and learning [27]. Children who experience poor sleep quality often struggle with concentration, problem-solving, and academic performance. Studies have shown that insufficient or disrupted sleep can lead to difficulties in processing information and performing complex cognitive tasks, which are crucial for success in school [37]. In addition to cognitive impairment, poor sleep quality is linked to emotional dysregulation. Children with inadequate sleep are more likely to exhibit symptoms of anxiety, depression, irritability, and mood swings, making it challenging to interact socially and maintain relationships [38]. Sleep deprivation affects the brain’s ability to regulate emotions, heightening stress responses, and reducing the ability to cope with everyday challenges [9]. Moreover, the lack of restorative sleep can weaken immune function, making children more susceptible to infections and chronic illnesses [12]. Sleep disturbances also interfere with the regulation of hormones that control appetite, such as leptin and ghrelin, increasing the risk of childhood obesity [28]. Beyond these health issues, poor sleep quality in children has been associated with long-term developmental consequences. Chronic sleep deprivation during childhood can have lasting impacts on brain development, affecting emotional and behavioral patterns well into adolescence and adulthood [37]. Given the extensive range of negative outcomes associated with poor sleep quality, early interventions that promote healthy sleep habits are crucial for ensuring the holistic well-being of children.

Impact of mobile phone addiction on sleep quality

Mechanisms of Influence

Mobile phone addiction significantly impacts sleep quality through multiple mechanisms, most notably through increased screen time and the psychological effects of constant connectivity. One primary mechanism is the disruption of circadian rhythms caused by the blue light emitted from mobile phone screens. Blue light exposure, particularly before bedtime, suppresses melatonin production, the hormone responsible for regulating sleep-wake cycles, leading to delayed sleep onset and poor sleep quality [38]. Children and adolescents, who are heavy users of mobile phones, are especially susceptible to this disruption, as their circadian rhythms are still developing and more vulnerable to environmental influences [39].

Another mechanism through which mobile phone addiction influences sleep quality is through psychological arousal. The constant notifications, messages, and updates from social media, games, and other mobile applications create a heightened state of mental engagement, making it difficult to relax and prepare for sleep [40]. This mental stimulation often leads to prolonged wakefulness and difficulty falling asleep. Furthermore, mobile phone addiction is linked to increased levels of stress and anxiety, particularly the fear of missing out (FOMO) and social comparison, which can keep users mentally alert and disrupt sleep continuity [41]. Behavioral patterns associated with mobile phone addiction, such as the habit of checking phones during the night or using them as an alarm, can cause frequent awakenings and sleep fragmentation. The more a child is exposed to their phone at night, the more likely they are to develop irregular sleep patterns, contributing to daytime fatigue, irritability, and decreased academic performance [42]. These mechanisms create a feedback loop, where poor sleep quality exacerbates the symptoms of mobile phone addiction, leading to further disruption of sleep.

Another mechanism by which mobile phone addiction affects sleep quality is the alteration of bedtime routines and sleep hygiene practices. With mobile phones becoming integral to daily life, many individuals, particularly adolescents, have checking their phones right before bed. This not only prolongs wakefulness but also interrupts essential wind-down activities, such as reading or engaging in relaxing practices that help transition into sleep [6]. Poor sleep hygiene habits, such as using mobile phones in dark rooms or scrolling through stimulating content, can exacerbate difficulties in falling asleep. Engaging with emotionally charged content, such as social media posts or news, may further stimulate emotional responses, triggering stress or excitement that interferes with sleep onset.

The factor that aggravates sleep disruption is the social pressure to stay connected, especially among younger users. Adolescents may feel compelled to remain engaged with their peers through social media, messaging, or online gaming, often leading to late-night interactions that delay sleep [43]. This pressure is amplified by group chats, notifications, and FOMO, making it difficult to disengage and prioritize sleep. This constant need for connectivity can shift the natural sleep schedule, contributing to the prevalence of delayed sleep phase syndrome, where individuals fall asleep much later than they should and experience difficulty waking up on time [43]. Mobile phone addiction is linked to heightened mental health issues such as anxiety and depression, both of which can further exacerbate sleep problems. Studies have found that individuals who experience high levels of anxiety often use their phones as a distraction or coping mechanism, which in turn exacerbates the cycle of poor sleep [44]. This link between mental health and sleep quality creates a vicious cycle where mobile phone addiction leads to poor mental health, which further impairs sleep patterns, creating long-term health and well-being issues (Table 4) [44].

**Table 4:** Details of studies that suggest mobile phone addiction negatively impacts the sleep quality of children

Aspect	Details
Night-time mobile phone use [6]	Adolescents who used mobile phones at night had delayed sleep onset, shorter sleep duration, and poorer sleep quality.
Blue light exposure [6]	Blue light from mobile phones delayed melatonin production, leading to sleep difficulties and reduced sleep quality.
Addiction and mental health [5]	Excessive smartphone use was linked to poor sleep quality, insomnia, depression, and anxiety among university students.
Social media and gaming use [6]	Adolescents who used mobile phones for social media and gaming before bed had reduced sleep duration and efficiency.
Chronic sleep disruption [5]	Prolonged mobile phone use led to chronic sleep deprivation, reduced academic performance, and worse sleep patterns.
Psychological arousal [40]	Constant notifications and updates led to heightened mental engagement, prolonging wakefulness and disrupting sleep.
Fear of missing out (FOMO) [41]	Fear of missing social updates on mobile phones contributed to sleep fragmentation and increased nighttime awakenings.
Emotional dysregulation [44]	Sleep disturbances caused by mobile phone addiction were linked to emotional instability, irritability, and mood swings.
Physical health consequences [45]	Poor sleep quality due to mobile phone addiction increased the risk of obesity, weakened immune function, and growth problems.
Academic performance impact [46]	Mobile phone overuse, especially at night, led to reduced academic performance due to impaired cognitive function and memory consolidation.
Increased anxiety and stress [43]	Mobile phone addiction was linked to higher levels of stress and anxiety, disrupting the sleep cycle and increasing the likelihood of sleep disturbances.
Bedtime routine disruption [6]	Using mobile phones before sleep delayed bedtimes, disrupted relaxation routines, and caused irregular sleep patterns.
Nomophobia [16]	Fear of being without a mobile phone (nomophobia) led to heightened nighttime usage, causing further sleep disruption and fragmented sleep.
Cognitive impairment [27]	Sleep disruptions caused by mobile phone overuse were linked to cognitive impairments such as poor attention, memory, and learning abilities in children.
Sleep-wake cycle alteration [38]	Blue light exposure and late-night usage shifted circadian rhythms, causing delayed sleep phase syndrome and difficulties waking up in the morning.
Musculoskeletal problems [47]	Extended mobile phone use in poor postures (e.g., in bed) contributed to musculoskeletal discomfort and pain, compounding physical health problems.
Sleep fragmentation [42]	Frequent checking of mobile phones during the night led to fragmented sleep and daytime fatigue, reducing overall sleep efficiency.
Aggressive and impulsive behavior [48]	Chronic sleep deprivation due to mobile phone overuse was linked to increased aggressive behavior and impulsivity in adolescents.

Variability of Impact

The impact of mobile phone addiction on sleep quality can vary significantly across individuals, influenced by factors such as age, gender, psychological health, and specific patterns of mobile phone use. Age is a key variable, with younger children and adolescents being more vulnerable to the negative effects of mobile phone addiction on sleep due to their developing circadian rhythms and higher susceptibility to the overstimulation caused by digital media [15]. Adolescents, in particular, are more prone to using mobile phones late into the night and engaging with social media, games, or messaging apps, which leads to delayed sleep onset and decreased sleep duration. Younger children, while less likely to be engaged with social media, may still suffer from sleep disturbances due to the addictive nature of mobile games and videos.

Gender differences also play a role in the variability of impact. Some studies have found that boys are more likely to use mobile phones for gaming, which has been associated with more severe sleep disturbances, whereas girls tend to use their phones for social communication, which may lead to anxiety and FOMO, further disrupting sleep quality [49]. Additionally, psychological factors such as anxiety, depression, and emotional regulation contribute to individual differences in the impact of mobile phone addiction. Individuals who experience higher levels of anxiety or emotional instability are more likely to use their phones as a coping mechanism, which can exacerbate sleep issues through increased psychological arousal.

The intensity of mobile phone use, including the duration and purpose of usage, also affects the degree of sleep disruption. Those who use their phones for social media and interactive activities (e.g., texting, gaming) report poorer sleep quality than those who use their phones for passive activities, such as listening to music or reading [49]. Environmental factors, including household rules about screen time and parental monitoring, further influence the extent of mobile phone addiction's impact on sleep. In households with strict rules regarding mobile phone use at bedtime, children are less likely to experience severe sleep disturbances [49].

Moreover, socioeconomic status (SES) can also influence the variability of the impact of mobile phone addiction on sleep quality. Research has shown that individuals from lower SES backgrounds may experience higher levels of mobile phone addiction due to increased stress, limited access to alternative recreational activities, and fewer resources for structured routines, including healthy sleep habits [21]. In such households, children may rely more heavily on mobile phones for entertainment or as a distraction from their environment, leading to increased screen time and more severe sleep disruptions. Parental involvement and education levels are crucial factors; parents with higher education levels and a better understanding of the health implications of excessive screen use may enforce stricter screen time limits, thus mitigating some of the negative effects of mobile phone addiction on sleep [6]. Conversely, in environments where screen use is less regulated, children may experience greater freedom in using mobile devices, exacerbating the risk of sleep disturbances. Cultural factors can also play a role, as different cultures place varying levels of importance on technology use, sleep routines, and family structure, all of which contribute to the degree of sleep disruption caused by mobile phone addiction [43].

Health implications of disrupted sleep due to mobile phone addiction

Cognitive and Behavioral Consequences

Disrupted sleep due to mobile phone addiction has significant cognitive and behavioral consequences, especially in children and adolescents. One of the most pronounced effects is on cognitive performance. Poor sleep quality and sleep deprivation are associated with deficits in attention, memory, and executive function, which are critical for learning and academic success [27]. Studies have shown that adolescents with high levels of mobile phone use, particularly at night, experience impaired ability to focus, process information, and solve problems. Sleep disruption hinders the brain’s ability to consolidate memories and optimize cognitive functions during the sleep cycle, leading to poorer academic performance and reduced ability to retain information learned during the day [46].

Behaviorally, disrupted sleep resulting from excessive mobile phone use has been linked to increased emotional instability, irritability, and impulsivity. Children and adolescents with insufficient sleep are more likely to exhibit hyperactivity, aggression, and difficulties with emotional regulation [48]. Mobile phone addiction, particularly the constant engagement with social media and games, can overstimulate the brain, leading to heightened arousal that disrupts sleep cycles, and as a result, affects daytime behavior. Sleep-deprived children often exhibit increased risk-taking behaviors and are more prone to mood swings and anxiety [40]. Additionally, mobile phone use can lead to higher stress levels due to factors like FOMO or social comparisons, exacerbating emotional difficulties and further contributing to sleep disturbances [44].

Chronic sleep disruption caused by mobile phone addiction can have long-term effects on mental health, particularly increasing the risk of developing anxiety and depression. Prolonged sleep deprivation negatively affects the brain’s ability to regulate emotions, leading to heightened emotional sensitivity and reduced resilience to stress [43]. This emotional dysregulation, compounded by the constant connectivity and overstimulation from mobile devices, can make adolescents more vulnerable to mental health disorders. Furthermore, the lack of restorative sleep impairs the brain’s stress-response system, increasing cortisol levels, which contributes to heightened anxiety and depressive symptoms [44]. These mental health issues, in turn, reinforce mobile phone dependency, creating a vicious cycle of poor sleep and deteriorating emotional health. Over time, the compounded effects of cognitive impairment and emotional instability may lead to more serious behavioral problems, such as social withdrawal, academic underperformance, and difficulties in interpersonal relationships [48]. Early interventions are crucial to break this cycle and promote healthier mobile phone usage and sleep habits in children and adolescents.

Emotional and Psychological Effects

The emotional and psychological effects of disrupted sleep due to mobile phone addiction are profound, particularly among children and adolescents. One of the most significant consequences is the increased risk of developing anxiety and depression. Studies have shown that prolonged exposure to mobile devices, especially social media and messaging apps, can lead to emotional distress, as children are constantly exposed to social comparison, cyberbullying, and FOMO [44]. These factors contribute to heightened anxiety and depressive symptoms, which are exacerbated by poor sleep quality. Sleep deprivation disrupts the brain's ability to regulate emotions, leading to increased emotional volatility, irritability, and a diminished ability to cope with stress [43].

Poor sleep resulting from excessive mobile phone use has been linked to heightened levels of loneliness and social isolation. Children and adolescents who spend excessive amounts of time on their phones, particularly late at night, often substitute meaningful face-to-face interactions with virtual communication, which can lead to feelings of isolation and loneliness [50]. This social withdrawal, compounded by poor sleep, further deteriorates mental health, creating a vicious cycle of emotional instability. Moreover, psychological stress caused by constant connectivity and overstimulation from mobile devices can also result in heightened levels of aggression and impulsivity. Studies indicate that children with disrupted sleep patterns due to mobile phone addiction are more likely to exhibit behavioral issues such as hyperactivity and aggressive behaviors [40]. The inability to disengage from technology and relax before sleep keeps the brain in a heightened state of arousal, which impairs emotional regulation and leads to these behavioral consequences.

The relationship between disrupted sleep and emotional health is bidirectional, meaning that while mobile phone addiction contributes to poor sleep and emotional distress, pre-existing emotional and psychological issues can also drive individuals to excessive mobile phone use. Adolescents who struggle with anxiety, depression, or low self-esteem may turn to their phones as a coping mechanism, seeking solace in virtual interactions, entertainment, or distractions [6]. However, this reliance on digital engagement can exacerbate their emotional struggles, as the overstimulation from constant screen exposure further disrupts their sleep, leading to a decline in mental health. Over time, this cycle of dependency can contribute to more serious mental health conditions, including chronic stress, mood disorders, and increased risk of substance abuse [44]. In addition, the pervasive nature of social media creates an environment of continuous social comparison, where adolescents may feel inadequate or left out, further aggravating their feelings of loneliness and social isolation [41]. Breaking this cycle requires addressing both sleep hygiene and emotional health through targeted interventions, such as setting limits on screen time, promoting face-to-face interactions, and implementing mental health support strategies in schools and homes.

Physical Health Implications

The wide-ranging effects of sleep disruption brought on by mobile phone addiction on physical health are especially troubling for children and teenagers. The effect on the immune system's performance is among the most important ones. The body needs sleep to renew and heal itself, and persistent sleep deprivation impairs immunity, increasing a person's susceptibility to infections and diseases [51]. Due to weakened immune systems, children who use their phones late at night and have poor sleep quality are more susceptible to recurrent colds, the flu, and other common illnesses.

An additional noteworthy consequence for physical health is the elevated likelihood of obesity. Research has indicated that a lack of sleep interferes with the regulation of hormones like ghrelin and leptin which influence hunger. Lack of sleep causes a drop in the hormone leptin, which indicates fullness, and an increase in the hormone ghrelin, which stimulates hunger and causes overeating and weight gain [52]. Additionally, kids who are dependent on their phones for entertainment often spend more time inactively staring at screens, which diminishes possibilities for exercise and raises the risk of obesity even more [45]. Long-term metabolic diseases including type 2 diabetes and cardiovascular disease can be predisposed to by these patterns.

**Figure 3:**
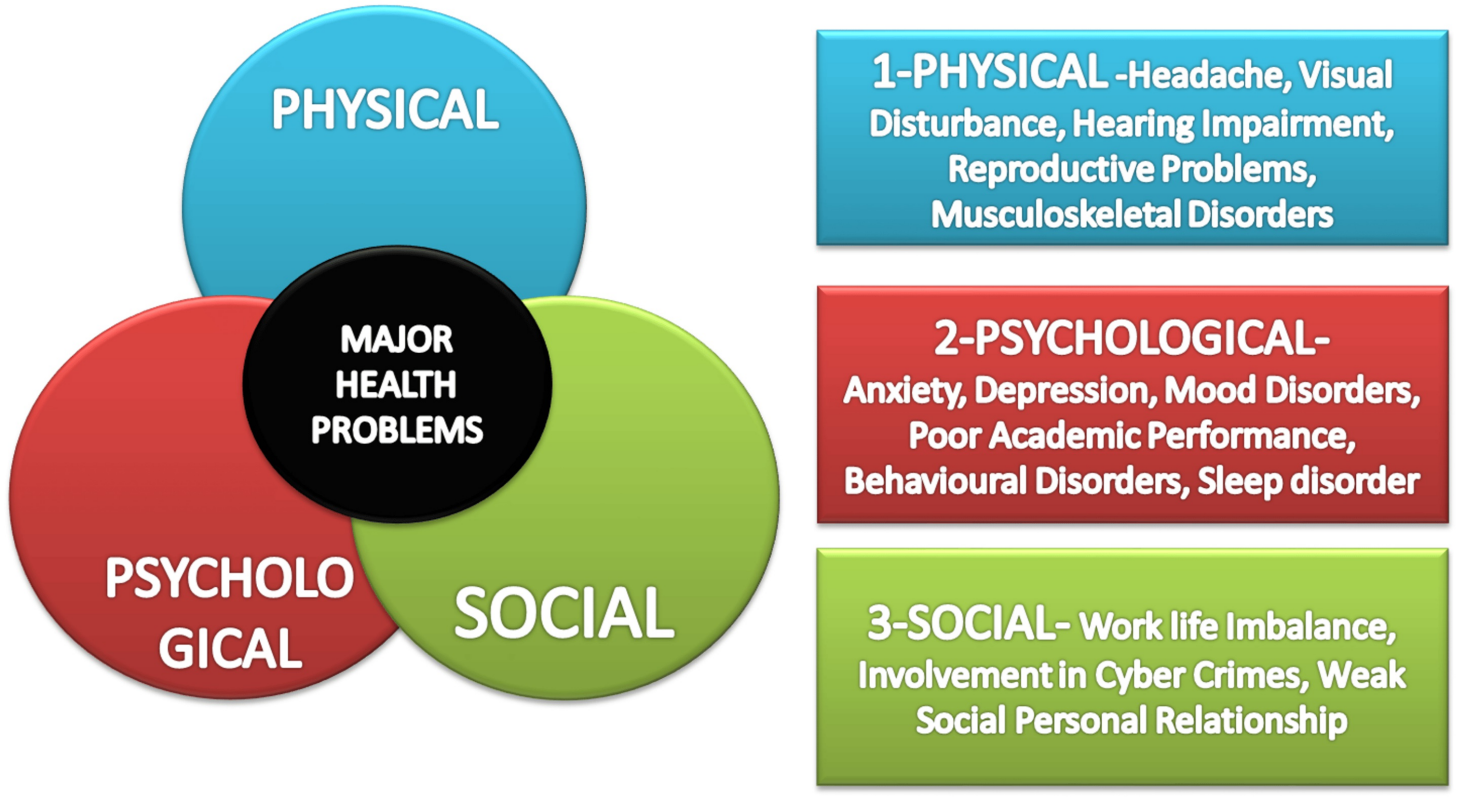
Physical and mental implications of mobile phone addiction Image credit: Created by Fatima G using Microsoft PowerPoint (Microsoft Corp., Redmond, WA, USA).

Children's growth and development are also impacted by sleep disruption caused by cell phone addiction. Growth hormones, which are essential for muscular development and physical growth, are released by the body during deep sleep stages [53,54]. Sleep disturbances can obstruct this process, which may result in stunted growth and developmental problems. According to Eitivipart et al. (2018) [47], extended screen use in incorrect settings is linked to poor posture and musculoskeletal discomfort, especially in the neck and back, when sleep loss occurs (Figure 3).

Interventions and recommendations

Strategies for Reducing Mobile Phone Addiction

Reducing mobile phone addiction, especially among adolescents, requires a multifaceted approach that combines education, behavior modification, and technological tools. One key strategy is raising awareness about the negative impacts of excessive phone use, such as its effects on sleep, mental health, and productivity. Educational programs, both in schools and at home, can teach children and adolescents how to recognize the signs of addiction and implement healthier digital habits. Setting clear boundaries around mobile phone usage is essential, such as designated screen-free times, particularly during meals, homework, and before bedtime. Parental controls and monitoring can help limit screen time, while apps designed to track usage or enforce time limits can provide additional support. Encouraging face-to-face interactions, outdoor activities, and hobbies that do not involve screens is another effective way to reduce dependency on mobile phones and promote healthier lifestyles [55].

Improving Sleep Quality

Improving sleep quality in children and adolescents impacted by mobile phone addiction requires the establishment of consistent sleep routines and healthy sleep hygiene practices. One of the most effective interventions is limiting screen time in the evening, especially within an hour or two before bedtime. Reducing exposure to blue light emitted by phones, which interferes with the production of melatonin, is critical for maintaining a healthy sleep-wake cycle. Creating a relaxing pre-sleep routine, such as reading, listening to calming music, or practicing mindfulness, can help signal to the brain that it is time to wind down. Ensuring that bedrooms are conducive to sleep dark, quiet, and free of electronic devices can also improve sleep quality. Additionally, maintaining a regular sleep schedule, where bedtimes and wake times are consistent, even on weekends, supports better sleep habits and reduces the negative effects of mobile phone overuse [56].

Policy Implications

Addressing mobile phone addiction and its impact on sleep quality requires not only individual and family-level interventions but also broader policy measures. Governments and educational institutions can play a significant role in promoting digital literacy and healthy mobile phone use. Schools can incorporate lessons on the responsible use of technology into their curricula, encouraging students to recognize the risks of overuse. Policies that regulate screen time, especially in educational settings, can also help mitigate the effects of excessive phone use. Public health campaigns can raise awareness about the consequences of mobile phone addiction, targeting both parents and children with practical advice and resources. Additionally, policymakers may consider advocating for tech companies to integrate features that promote responsible usage, such as screen time monitoring, automatic nighttime mode, or digital wellness tools that remind users to take breaks. These efforts can create a more supportive environment for reducing mobile phone addiction and improving overall well-being [57].

Critical analysis and research gaps

Limitations of Current Research

Current research on mobile phone addiction and its impact on sleep quality, while extensive, faces several limitations. One of the primary limitations is the reliance on self-reported data, particularly in studies involving adolescents. Self-reported screen time and sleep patterns can often be inaccurate, as individuals may underreport their usage or be unaware of the exact impact their behaviors have on their sleep quality. Another limitation is the cross-sectional nature of many studies, which makes it difficult to establish causality. While studies have shown a correlation between mobile phone addiction and poor sleep, it remains unclear whether phone addiction causes sleep problems or whether individuals with pre-existing sleep issues are more prone to excessive phone use. Additionally, most studies focus on short-term impacts, with fewer longitudinal studies that examine the long-term consequences of mobile phone addiction on sleep quality and overall health. Finally, there is a lack of standardized definitions and measurements of mobile phone addiction, making it challenging to compare results across different studies and fully understand the scope of the issue [58].

Areas for Future Research

Future studies could fill many of the knowledge gaps that now exist on cell phone addiction and its consequences. Initially, longitudinal research is required to investigate the long-term effects of mobile phone addiction on mental and physical health as well as sleep quality. The directionality of the link between phone addiction and sleep problems may be made clearer by these investigations. Second, more objective techniques for gauging the quality of sleep and the use of mobile phones should be developed in future study. One such technique would be to combine sleep-monitoring gadgets with usage-tracking applications. This would lessen the dependency on self-reports and produce data that is more accurate. Examining the social and cultural determinants of mobile phone addiction is a crucial topic for further study. The majority of research is focused on wealthy nations, which leaves a vacuum in our knowledge of how these problems impact people in less developed or economically challenged areas. Furthermore, studies might examine how well different treatments like behavioral therapies and digital literacy initiatives work to lessen phone addiction and enhance the quality of sleep [59].

Theoretical Perspectives

Theoretical perspectives in the study of mobile phone addiction have largely focused on psychological and behavioral frameworks, such as the addiction model and the theory of planned behavior. The addiction model suggests that mobile phone addiction exhibits characteristics similar to substance abuse, including withdrawal symptoms, tolerance, and negative consequences on daily functioning. This framework has been useful in identifying the compulsive nature of phone use but may not fully capture the social and cultural dimensions of the behavior [60]. The theory of planned behavior, which emphasizes the role of attitudes, social norms, and perceived behavioral control, helps explain why individuals may engage in excessive phone use despite knowing its negative consequences. However, more integrative approaches that combine psychological, sociological, and technological perspectives are needed to fully understand the complexity of mobile phone addiction. Future theoretical work could also explore the role of neurobiological factors, such as how mobile phone use stimulates reward pathways in the brain, reinforcing addictive behaviors. Additionally, examining the impact of digital environments and design choices in mobile applications on user behavior could provide a more holistic view of the factors driving mobile phone addiction [60].

## Conclusions

The impact of mobile phone addiction on the sleep quality of children is a growing public health concern. The addictive nature of mobile devices, particularly through their accessibility and the instant gratification they offer, has significant consequences on children’s physical, emotional, and cognitive well-being. This study has highlighted the strong connection between excessive mobile phone use and poor sleep quality, driven primarily by disruptions in circadian rhythms and heightened psychological arousal. The blue light emitted by screens, coupled with the compulsive need to stay connected through social media or gaming, leads to delayed sleep onset, reduced sleep duration, and overall sleep fragmentation. These disturbances not only affect immediate cognitive performance and emotional regulation but also contribute to long-term developmental issues, including the risk of obesity, weakened immune function, and behavioral problems. Addressing mobile phone addiction in children requires a multifaceted approach that includes educational programs, parental monitoring, and policy interventions aimed at promoting healthier digital habits and improving sleep hygiene. Early interventions are crucial in preventing the long-term adverse effects associated with poor sleep quality, thereby ensuring the holistic well-being of children. Future research should focus on longitudinal studies to better understand the long-term impact of mobile phone addiction on sleep and overall health, as well as the effectiveness of various interventions to mitigate this growing problem.
